# Functional brain abnormalities in patients with somatic symptom disorder presenting with chest pain: a resting-state fMRI study

**DOI:** 10.1038/s41598-026-51822-2

**Published:** 2026-05-16

**Authors:** Yi-Fan Ding, Ke-Ying Wang, Ting Yang, Xue-Fei Liu, Ying Li, Hai-Feng Shi, Jin-Wei Qiang

**Affiliations:** Department of Radiology, Jinshan Hospital, Fudan University, Shanghai, 201508 China

**Keywords:** Diseases, Medical research, Neurology, Neuroscience

## Abstract

Somatic symptom disorder (SSD) is characterized by persistent physical symptoms that cannot be fully explained by structural abnormalities or laboratory findings. However, the underlying neural mechanisms of SSD remain poorly understood. This study aimed **t**o investigate functional brain abnormalities in SSD patients presenting predominantly with chest pain using resting-state functional magnetic resonance imaging (rs-fMRI). A total of 102 participants were prospectively enrolled, including 56 patients with SSD and 46 healthy controls (HCs). All participants underwent clinical assessments and structural MRI examinations, including T2-weighted imaging (T2WI), T1-weighted imaging (T1WI), diffusion-weighted imaging (DWI), fluid-attenuated inversion recovery (FLAIR), and rs-fMRI. The rs-fMRI metrics included regional homogeneity (ReHo), amplitude of low-frequency fluctuations (ALFF), and fractional ALFF (fALFF). Group comparisons were conducted to identify differences in brain activity, and correlation analyses were performed to examine associations between brain activity and clinical symptoms. No significant differences were observed between the two groups in demographic variables, including age, gender, and education level. Clinical assessments revealed that SSD patients scored significantly higher than HCs on the Patient Health Questionnaire-15 (PHQ-15), Somatic Symptom Scale (SSS), Generalized Anxiety Disorder-7 (GAD-7), and Hamilton Depression Scale (HAMD). ALFF, fALFF, and ReHo analyses demonstrated significant alterations in spontaneous neural activity in SSD patients compared with HCs. Specifically, increased activity was observed in the left inferior frontal gyrus and precuneus, whereas decreased activity was detected in the hippocampus and insula. Correlation analyses revealed significant associations between these abnormal brain activity patterns and clinical symptom severity in SSD patients. This study highlights regional functional brain abnormalities in SSD, particularly in regions associated with emotion regulation, memory, and sensory processing. These findings provide novel insights into the neural mechanisms underlying SSD and suggest potential neuroimaging targets for therapeutic interventions that may help alleviate symptoms and improve patient outcomes.

**Supplementary Information:**

The online version contains supplementary material available at 10.1038/s41598-026-51822-2.

## Introduction

Somatic symptom disorder (SSD) is a complex psychiatric condition characterized by excessive preoccupation with distressing physical symptoms that cannot be fully explained by identifiable medical causes^1^. SSD often involves pain, chest discomfort, gastrointestinal distress, or fatigue, and is accompanied by high levels of anxiety and maladaptive thoughts about bodily sensations. SSD is associated with significant functional impairment and increased health care utilization, imposing a heavy socioeconomic burden^2^. Conceptually, SSD represents a disorder of perception and regulation, in which physiological sensations are misinterpreted and amplified through emotional and cognitive processes.

Previous studies have indicated that the brain’s ability to sense and interpret internal bodily signals is a key mechanism underlying SSD^3^. The interoceptive system involves a network encompassing the insula, anterior cingulate cortex (ACC), somatosensory cortex, and prefrontal regions, which collectively integrate sensory, affective, and cognitive information about bodily states^4^. Dysregulated interoceptive processing can lead to heightened self-focus on bodily sensations and the misattribution of normal physiological changes as pathological. Recent theories emphasize that SSD may stem from a failure to appropriately integrate emotional and bodily information, resulting in excessive prediction errors within interoceptive networks^5^. This framework provides a neurobiological explanation for how emotional dysregulation contributes to the persistence and amplification of physical symptoms.

Functional magnetic resonance imaging (fMRI) studies have provided increasing insight into these mechanisms, revealing alterations across multiple large-scale brain networks in SSD. Among fMRI analytical metrics, regional homogeneity (ReHo) quantifies local synchronization of neural activity, reflecting the temporal coherence of neighboring voxels; amplitude of low-frequency fluctuations (ALFF) measures the intensity of spontaneous neural oscillations; and fractional ALFF (fALFF) represents a normalized index of spontaneous activity relative to the entire frequency range, thereby reducing physiological noise^6^. fMRI research has consistently identified abnormalities within the salience network, particularly the anterior insula and ACC, regions crucial for integrating emotional and interoceptive signals^7^. Studies have reported increased resting-state functional connectivity between the insula and limbic structures such as the amygdala and hippocampus, as well as disrupted connectivity between the default mode network and executive control networks^8^. These findings suggest that SSD involves a functional imbalance between bottom-up interoceptive signaling and top-down cognitive control mechanisms. Structural neuroimaging studies have reported abnormalities in brain regions involved in interoception, emotion regulation, and salience processing in patients with somatic symptom disorders^9^. Specifically, increased ALFF in the medial prefrontal cortex and decreased fALFF in the precuneus reflect a functional dissociation within the default mode network, which may underlie exaggerated self-referential processing and maladaptive rumination about bodily states^10^.

However, prior studies have yielded inconsistent findings, partly due to methodological heterogeneity, small sample sizes, and the inclusion of patients with mixed somatic symptoms or psychiatric comorbidities^11^. Moreover, most imaging studies have treated SSD as a unitary condition, thereby neglecting possible neural distinctions among symptom-specific subtypes. Given that different symptom profiles may engage distinct sensory and emotional circuits, investigating symptom-specific subtypes is crucial for delineating the disorder’s pathophysiology. To date, limited research has focused specifically on SSD patients presenting with cardiopulmonary symptoms, such as chest pain and tightness, which represent one of the most prevalent and distressing clinical manifestations of SSD.

Based on prior evidence, we hypothesized that SSD patients would exhibit altered activity in the insula, anterior cingulate cortex, hippocampus, and prefrontal regions, reflecting disrupted interoceptive and emotion regulation networks. Specifically, we predicted increased ReHo and ALFF in prefrontal and limbic regions associated with hypervigilance and affective processing, alongside decreased neural activity in sensory integration regions, such as the insula and somatosensory cortex. By clarifying how altered brain activity relates to symptom severity and emotional dysregulation, this study aims to refine the neurobiological model of SSD, offering a foundation for personalized, brain-based diagnostic and therapeutic strategies.

## Methods

### Ethical approval

This prospective study received approval from the Ethics Committee of Jinshan Hospital, Fudan University (approval number: JIEC-2025-S09). Written informed consent was obtained from all participants prior to their inclusion in the study. The study protocol strictly adhered to the principles outlined in the Declaration of Helsinki.

### Study design and sample size estimation

This study employed a prospective, cross-sectional design to investigate functional brain abnormalities in patients with SSD presenting predominantly with chest pain. Resting-state fMRI (rs-fMRI) was used to assess regional spontaneous brain activity differences between SSD patients and healthy controls (HCs). The SSD group consisted of individuals diagnosed according to the Diagnostic and Statistical Manual of Mental Disorders, Fifth Edition (DSM-5) criteria, while HCs were matched by age, gender, and education level. Comprehensive neurocognitive and clinical assessments were conducted to evaluate somatic, anxiety, and depressive symptoms, while ensuring the exclusion of individuals with severe psychiatric or neurological disorders.

The sample size was comparable to that reported in previous rs-fMRI studies examining ALFF, fALFF, and ReHo alterations between patient groups and healthy controls, which typically include sample sizes in the range of 30–40 participants per group. Post-hoc power analyses conducted using G*Power 3.1 confirmed that the achieved sample size (56 SSD patients, 46 controls) provided adequate statistical power (> 0.90) to detect medium-to-large between-group effects (Cohen’s d ≥ 0.60) at α = 0.05 (two-tailed). However, several clusters yielded smaller effect sizes (Cohen’s d = 0.36–0.43), for which the present sample may have been underpowered. Correlation analyses between brain activity indices and clinical scales demonstrated statistical power ranging from 0.74 to 0.86 for observed effect sizes (*r* = 0.30–0.37).

### Participants

From 15 March 2025 to 15 July 2025, this study recruited 64 right-handed individuals experiencing predominant symptoms of chest pain, with or without additional somatic complaints, from the Neurology Clinic of Jinshan Hospital, Fudan University. Diagnoses were established by neurologists, including at least two attending physicians, one of whom was a deputy chief physician, using the DSM-5 diagnostic criteria. A diagnosis of SSD was confirmed when the Patient Health Questionnaire-15 (PHQ-15) score was greater than 5.

Inclusion criteria for SSD were as follows: (1) age between 19 and 70 years; (2) Mini Mental State Examination (MMSE) score greater than 24, indicating no significant cognitive or intellectual impairment; (3) fulfillment of DSM-5 diagnostic criteria for SSD, with chest pain or tightness as the main symptoms and no other dominant somatic complaints; (4) no use of psychotropic medications within the preceding two months; (5) no contraindications for MRI scanning; (6) first-episode patients with SSD with no prior pharmacological or psychological treatment; (7) all SSD participants underwent comprehensive cardiopulmonary evaluations, including electrocardiography, echocardiography, and chest computed tomography, to rule out ischemic heart disease, structural cardiac abnormalities, or pulmonary pathology.

Exclusion criteria for SSD were as follows: (1) current or past history of severe neurological or systemic medical diseases; (2) current or past history of psychiatric disorders, psychoactive substance use, or alcohol dependence.

A total of 46 healthy volunteers matched for age, gender, and education level were recruited as HCs. Inclusion criteria for HCs were: (1) age between 19 and 70 years; (2) normal cognitive function; (3) right-handedness; (4) no contraindications for MRI scanning; (5) no history of systematic medication use or psychotherapy. Exclusion criteria for HCs included: (1) any current or past diagnosis meeting DSM-5 criteria for psychiatric disorders; (2) current or past history of severe neurological or organ diseases; (3) the presence of clinically significant anxiety or depressive symptoms, leading to exclusion from the control group.

### Clinical and neurocognitive measures

All participants completed the MMSE, PHQ-15, Somatic Symptom Scale (SSS), Generalized Anxiety Disorder-7 (GAD-7), and Hamilton Depression Scale (HAMD) to assess global cognitive function, somatic symptom severity, anxiety, and depressive symptoms, respectively.

### MRI acquisition

MRI data were acquired using a 3.0 T scanner (Verio, Siemens, Erlangen, Germany) at the Department of Radiology, Jinshan Hospital. Participants wore noise-reducing earplugs, and foam padding was applied to minimize head motion. Prior to scanning, detailed instructions were provided, and participants were instructed to remain awake, relaxed, and motionless during image acquisition. Structural images, including a high-resolution three-dimensional T1-weighted MPRAGE sequence (T1WI, providing an anatomical reference for spatial normalization), T2-weighted imaging (T2WI), diffusion-weighted imaging (DWI), and fluid-attenuated inversion recovery (FLAIR), were acquired first to rule out organic brain lesions or neurodegenerative changes. Resting-state functional images were subsequently obtained using a 32-channel head coil with the following parameters: repetition time (TR) = 3,000 ms, echo time (TE) = 45 ms, flip angle = 90°, interslice gap = 0.75 mm, number of slices = 36, field of view (FOV) = 240 × 240 mm², and slice thickness = 3 mm. Each scanning session lasted 600 s, yielding 200 time points per participant.

### Data preprocessing

Data preprocessing was performed using SPM12 (implemented in MATLAB; https://www.fil.ion.ucl.ac.uk/spm/software/spm12/) and DPABI 3.0 (https://rfmri.org/DPABI), also implemented in MATLAB. DICOM images were first converted to NIfTI format using SPM12. The initial 10 volumes were discarded to allow for signal stabilization. Slice timing correction and realignment were applied to correct for temporal delays and head motion. Participants with head motion exceeding 3 mm or 3° were excluded. Additionally, the proportion of volumes with mean framewise displacement (FD) > 0.5 mm was assessed as an auxiliary motion metric. Functional images were normalized to the Montreal Neurological Institute (MNI) EPI template and resampled to voxels of 3 × 3 × 3 mm³. Nuisance covariates (including head motion parameters, white matter signals, and cerebrospinal fluid signals) were regressed out, and linear trends were removed.

### ReHo, ALFF, and fALFF analyses

ReHo was calculated on unsmoothed preprocessed functional images to avoid artificially increasing local synchronization. Temporal band-pass filtering (0.01–0.1 Hz) was applied only for the ReHo analysis to reduce physiological noise and low-frequency drift. After ReHo maps were generated using Kendall’s coefficient of concordance among each voxel and its 26 neighboring voxels, spatial smoothing with a Gaussian kernel (FWHM = 6 mm) was applied to the resulting ReHo maps prior to group-level statistical analysis. ReHo values were standardized by z-score normalization within a gray matter mask derived from individual T1-weighted segmentation, thereby minimizing contamination from white matter and cerebrospinal fluid. Two-sample *t*-tests were performed in SPM12, with age and gender as covariates.

ALFF and fALFF analyses were conducted using the DPABI toolbox on temporally unfiltered preprocessed data. After metric calculation, spatial smoothing with a Gaussian kernel (FWHM = 6 mm) was applied to the resulting ALFF and fALFF maps to improve the signal-to-noise ratio and facilitate group-level statistical analyses. Between-group comparisons were performed using two-sample *t*-tests in SPM12, with age and gender as covariates.

### Correlation analysis

Mean ReHo, ALFF, and fALFF values were extracted from clusters surviving multiple-comparison correction (voxel-wise *p* < 0.001, cluster-wise *p*_FWE < 0.05) using the region-of-interest (ROI) extraction function in DPABI. Correlations between neuroimaging metrics and clinical scores were analyzed using GraphPad Prism. Normality was assessed in SPSS, and Pearson or Spearman correlation coefficients were calculated accordingly. *P*-values were adjusted using the Benjamini–Hochberg false discovery rate (FDR) correction (*q* < 0.05). Only correlations that remained significant after correction were reported.

To account for the potential confounding effects of psychiatric comorbidities, partial correlation analyses were performed with GAD-7 and HAMD scores as covariates to determine whether the associations between fMRI indices and somatic symptom severity (PHQ-15 and SSS) remained significant.

### Statistical analyses

Statistical analyses were performed to evaluate differences in demographic, clinical, and neuroimaging variables between SSD patients and HCs. Group differences in demographic and clinical variables were analyzed using two-sample *t*-tests, nonparametric tests, or chi-square tests, as appropriate. rs-fMRI measures (ReHo, ALFF, and fALFF) were compared using two-sample *t*-tests with age, gender and mean FD included as covariates.

Voxel-wise multiple comparisons were corrected using nonparametric permutation‑based family‑wise error (FWE) correction implemented in the SnPM13 toolbox within SPM12 (10,000 permutations), with a voxel-wise threshold of *p* < 0.001 (uncorrected) and a cluster-wise threshold of *p*_FWE < 0.05 (two-tailed). Sensitivity analyses were conducted using stricter head motion criteria (mean FD < 0.2 mm) and varying cluster-forming thresholds (*p* < 0.005 and *p* < 0.0005). Spatial overlap between the original and reanalyzed clusters was quantified to evaluate robustness.

Finally, voxel-wise multiple regression analyses were conducted using PHQ-15 and SSS scores as continuous predictors of ReHo, ALFF, and fALFF, while controlling for age, gender, and mean FD. Significant clusters were identified using the same permutation-based FWE correction.

## Results

### Demographic and clinical characteristics of the participants

A total of 102 individuals participated in the study, comprising 56 SSD patients and 46 HCs after excluding participants with excessive head motion. Demographic variables such as age, gender, and education level showed no significant differences between the two groups, indicating good comparability between them. Clinical assessments revealed that SSD patients scored significantly higher than HCs on the PHQ-15, SSS, GAD-7, and HAMD (all *p* < 0.05) (Table 1). Head motion parameters did not differ significantly between SSD patients and HCs (all *p* > 0.05). These motion-related data are summarized in Supplementary Table 1.

**Table 1:** Demographic and Clinical Characteristics and results of neurocognitive tests.

Parameters	SSD (*N* = 56)	HC (*N* = 46)	*P* value
Demographic variables			
Age, year	47 ± 12.1	42 ± 14.6	0.074
Sex, male/female	25/31	23/23	0.590
Education	10.8 ± 3.3	11.0 ± 3.8	0.757
Clinical variables			
MMSE	27.2 ± 1.2	27.7 ± 1.1	0.065
PHQ-15	8.3 ± 2.5	1.3 ± 0.7	< 0.001
SSS	35.4 ± 3.0	22.2 ± 0.7	< 0.001
GAD-7	5.3 ± 3.9	3.9 ± 1.5	< 0.004
HAMD	6.6 ± 2.9	4.1 ± 1.7	< 0.001

SSD, somatic symptom disorder; HC, healthy controls; MMSE, Mini-mental State Examination; PHQ-15, Patient Health Questionnaire-15; SSS, the somatic symptom scale; GAD-7, Generalized Anxiety Disoeder-7; HAMD, Hamilton Depression Scale.

### ReHo analysis

**Fig. 1:**
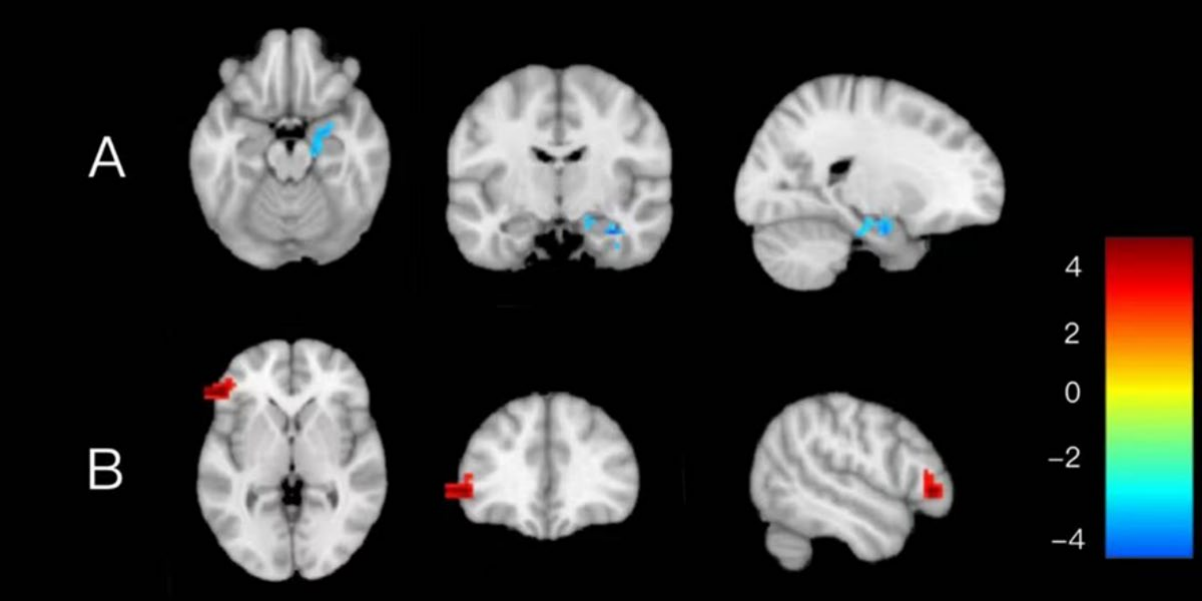
rs-fMRI results showing regions with significant ReHo differences between SSD patients and HCs. (**A**) Regions with decreased ReHo in SSD patients (blue), primarily involving the right hippocampus (HIP), associated with impaired memory and emotion regulation. (**B**) Regions with increased ReHo in SSD patients (red), mainly located in the left inferior frontal gyrus (IFG), triangular part, linked to heightened emotional and cognitive regulation. Axial, coronal, and sagittal views are displayed. Statistical thresholds were set at *P* < 0.001 (voxel-level) and pFWE < 0.05 (cluster-level). (rs-fMRI, Resting-state functional magnetic resonance imaging; ReHo, regional homogeneity; SSD, somatic symptom disorder; HCs, healthy controls; FWE, family-wise errors)

ReHo analysis revealed significant differences between SSD patients and HCs. The SSD group exhibited significantly elevated ReHo values in the left inferior frontal gyrus, a region implicated in emotion regulation and cognitive control. Conversely, significantly reduced ReHo values were observed in the right hippocampus, suggesting potential impairments in memory processing and emotional regulation among SSD patients (Table 2; Fig. 1).

**Table 2:** Regions showing ReHo, ALFF, and fALFF differences between groups.

Parameters	Brain regions	Left/Right	Number of voxels	Peak (MNI) x y z	Cohen’s d	*P* value
ReHo	SSD> controls					
	Inferior frontal gyrus, triangular part	Left	74	-51 33 0	0.43	0.016
	SSD< controls					
	Hippocampus	Right	130	27 − 6 -18	-0.63	0.001
ALFF	SSD> controls					
	Angular gyrus	Right	35	51–63 24	0.51	0.006
	Superior parietal gyrus	Left	42	-15 -72 48	0.59	0.002
	Middle frontal gyrus	Right	205	42 48 0	0.67	< 0.001
	Inferior temporal gyrus	Left	37	-54 -60 -6	0.54	0.004
	Superior medial frontal gyrus	Right	41	6 57 12	0.59	0.002
	Superior occipital gyrus	Right	25	30–84 27	0.36	0.036
	Precentral gyrus	Left	36	-51 6 21	0.52	0.005
	SSD< controls					
	Superior frontal gyrus	Right	111	21 − 12 60	-0.67	< 0.001
	Middle cingulate gyrus	Left	172	0–12 45	-0.67	0.000
	Paracentral lobule	Left	47	-9 -18 63	-0.63	0.001
fALFF	SSD> controls					
	Middle frontal gyrus	Right	20	39 36 24	0.51	0.006
	Superior medial frontal gyrus	Left	23	0 27 42	0.59	0.002
	Precuneus	Left	23	-9 -78 42	0.59	0.002
	SSD< controls					
	Insula	Left	15	-36 -18 6	-0.38	0.030

ReHo, regional homogeneity; ALFF, amplitude of low frequency fluctuations; fALFF, fractional amplitude of low frequency fluctuations; MIN, Montreal Neurological Institute.

*P* < 0.001 is considered significant, and at the cluster-level, after FWE (family-wise errors) correction; *P* < 0.05 is considered significant, indicating that the voxel is significantly different between the SSD patient group and the HC group.

### ALFF and fALFF analyses

**Fig. 2:**
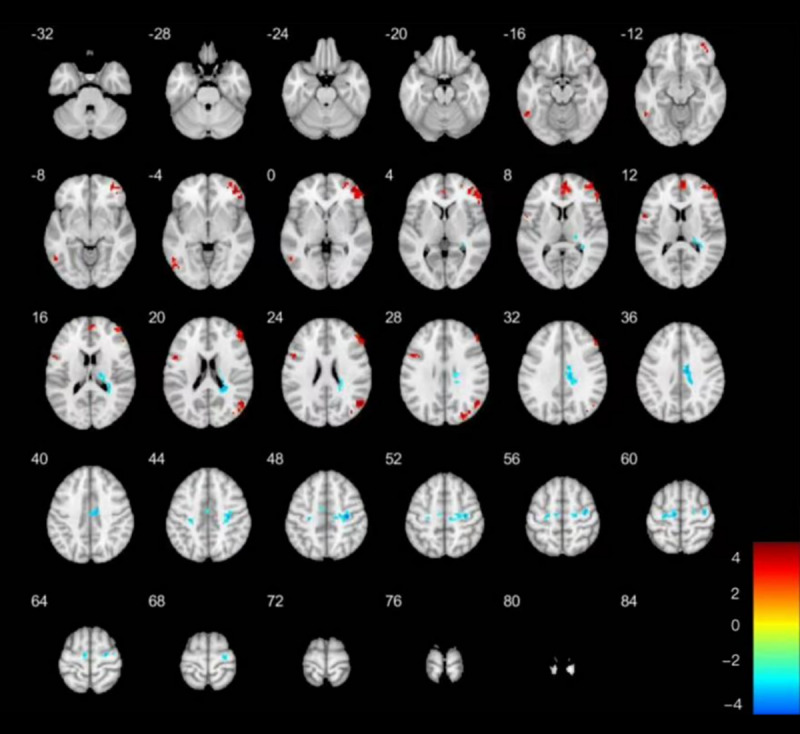
Axial brain slices illustrating ALFF differences between SSD patients and HCs. Compared with HCs, SSD patients exhibited significantly increased ALFF in the right angular gyrus (ANG), right middle frontal gyrus (MFG), right superior medial frontal gyrus (SFG.med), right superior occipital gyrus (SOG), left superior parietal gyrus (SPG), left inferior temporal gyrus (ITG), and left precentral gyrus (PreCG). Conversely, significantly decreased ALFF was observed in the right superior frontal gyrus (SFG), left middle cingulate gyrus (DCG), and left paracentral lobule (PCL). Numbers above each slice indicate the z-coordinate in MNI space. (ALFF, amplitude of low frequency fluctuations; MNI, Montreal Neurological Institute.)

ALFF and fALFF analyses identified marked differences in spontaneous neural activity between SSD patients and HCs. Significantly increased ALFF values were observed in the right angular gyrus, left superior parietal gyrus, right middle frontal gyrus, left inferior temporal gyrus, right superior medial frontal gyrus, right superior occipital gyrus, and left precentral gyrus in SSD patients. These regions are primarily associated with cognitive control, memory processing, and emotional regulation. Conversely, significantly reduced ALFF values were found in the right superior frontal gyrus, left middle cingulate gyrus, and left paracentral lobule, indicating diminished neural activity in regions linked to higher-order cognitive and motor functions (Table 2; Fig. 2).

**Fig. 3:**
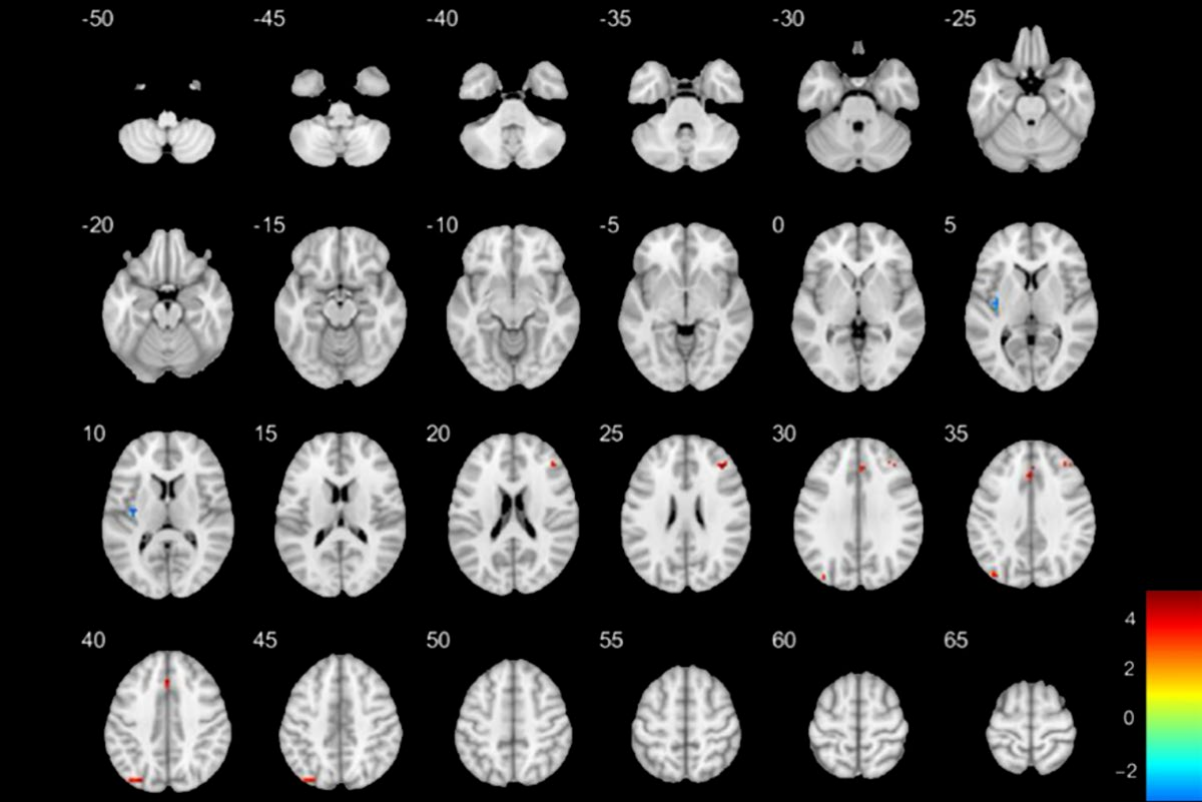
Axial brain slices showing fALFF differences between SSD patients and HCs. Compared with HCs, SSD patients exhibited significantly increased fALFF in the right middle frontal gyrus (MFG), left superior medial frontal gyrus (SFG.med), and left precuneus (PCUN), along with decreased fALFF in the left insula (INS). Numbers above each slice indicate the z-coordinates in MNI space. (fALFF, fractional amplitude of low frequency fluctuations.)

The fALFF analysis further demonstrated significantly increased activity in the right middle frontal gyrus, left superior medial frontal gyrus, and left precuneus, which is a core node of the default mode network involved in self-referential processing and memory. In contrast, significantly decreased fALFF values were observed in the left insula, a region critically involved in sensory integration and emotional awareness (Table 2; Fig. 3).

### Correlation analysis

After applying FDR correction for multiple comparisons, correlation analyses revealed significant associations between altered brain activity and clinical symptom severity in SSD patients (Supplementary Fig. 1). ReHo values in the right hippocampus showed a significant negative correlation with SSS scores (*r* = -0.36, *p* = 0.006, and *p*_FDR = 0.018). The correlation between fALFF in the left middle cingulate gyrus and paracingulate gyrus and GAD-7 scores showed a trend-level association (*p*_FDR = 0.081) but did not survive FDR correction. Furthermore, fALFF values in the left precuneus showed significant positive correlations with PHQ-15 scores (*r* = 0.36, *p* = 0.008, and *p*_FDR = 0.015) and SSS scores (*r* = 0.37, *p* = 0.006, and *p*_FDR = 0.019).

After controlling for anxiety and depressive symptom severity as measured by GAD-7 and HAMD scores, the correlations between right hippocampal ReHo and SSS (*r* = -0.32, *p* = 0.012), left precuneus fALFF and PHQ-15 (*r* = 0.34, *p* = 0.010), and left precuneus fALFF and SSS (*r* = 0.33, *p* = 0.011) remained statistically significant (Supplementary Table 2).

Voxel-wise regression analyses revealed that higher PHQ-15 and SSS scores were positively correlated with ALFF in the left precuneus and medial prefrontal cortex, and negatively correlated with fALFF in the left insula across all participants, with these associations surviving FWE correction (*p*_FWE < 0.05).

### Sensitivity analyses

Sensitivity analyses confirmed the robustness of the primary findings. When applying a stricter head motion criterion (mean FD < 0.2 mm), all major clusters, particularly the left inferior frontal gyrus, right hippocampus, and left insula-remained significant, exhibiting over 85% spatial overlap relative to the primary analysis. Similarly, the results remained stable across varying cluster-forming voxel-wise thresholds (Supplementary Table 3).

## Discussion

This study identified functional brain abnormalities in SSD patients with predominant chest pain, revealing altered spontaneous neural activity across emotion-regulatory, sensory, and interoceptive networks. Using ReHo, ALFF, and fALFF analyses, we observed increased activity in the left inferior frontal gyrus and precuneus, along with decreased activity in the hippocampus and insula. These alterations were associated with clinical symptom severity, suggesting a link between neural dysregulation and somatic symptom burden.

The observed ReHo, ALFF, and fALFF abnormalities reflect distinct yet interconnected neurobiological processes. ReHo changes indicate altered local synchronization within key regulatory hubs. Increased ReHo in the inferior frontal gyrus may reflect heightened local coordination related to cognitive monitoring and emotional inhibition^12^. Reduced ReHo in the right hippocampus was associated with greater somatic symptom severity, as reflected by higher SSS scores. The hippocampus is widely implicated in memory processing, contextual learning, and the regulation of emotional and stress-related responses, and it plays a central role in integrating contextual information with cognitive and affective processes^13^. From this perspective, altered intrinsic activity in the hippocampus may be related to differences in how individuals with SSD process or contextualize internal bodily sensations. A modest correlation indicates an association rather than a causal relationship between hippocampal intrinsic activity and somatic symptom severity.

In addition, hippocampal structure and function are known to be influenced by affective symptoms such as anxiety and depression. Previous studies have demonstrated hippocampal alterations in mood and anxiety disorders, suggesting potential overlap in neural mechanisms across these conditions^14^. Although anxiety and depression scores were statistically controlled in the present analysis, the SSD group exhibited elevated affective symptom levels compared with healthy controls, and residual confounding cannot be completely excluded. Future studies using longitudinal designs or larger samples will be necessary to further clarify the relationships among hippocampal function, affective symptoms, and somatic symptom severity in SSD.

ALFF and fALFF capture broader changes in spontaneous neural activity amplitude and signal specificity. Increased ALFF in prefrontal and parietal regions suggests amplified intrinsic neural oscillations, potentially reflecting excessive top-down cognitive engagement. The insula plays a central role in interoceptive processing and salience detection. Although increased insular activity has been associated with heightened interoceptive awareness and somatic symptom severity, the present finding of decreased fALFF may reflect altered baseline intrinsic activity in this region^15^.

Notably, prior studies of SSD have reported both hyperactivation and hypoactivation of interoceptive regions, particularly the insula and anterior cingulate cortex^16,17^. These divergent findings suggest that interoceptive dysregulation in SSD does not follow a uniform direction but instead reflects a context-dependent imbalance between bottom-up bodily signaling and top-down cognitive control. Our findings of insular hypoactivation alongside prefrontal hyperactivation may represent compensatory modulation of interoceptive processing under persistent symptom distress.

The increased precuneus fALFF observed in our cohort further supports this imbalance. The precuneus, a central node of the default mode network (DMN), is involved in self-referential processing and episodic memory. Its hyperactivation, positively correlated with symptom severity, may reflect maladaptive somatic rumination, failed DMN suppression at rest, or compensatory efforts to contextualize ambiguous bodily sensations. Given that fALFF does not capture connectivity patterns, increased local activity may coexist with disrupted network-level coordination, particularly between the DMN and executive control networks.

Overall, our findings are consistent with previous neuroimaging studies demonstrating insular, cingulate, and hippocampal abnormalities in SSD^18^, while extending them by identifying a dual pattern of hyperactivation in cognitive regions and hypoactivation in interoceptive regions. Similar dual patterns have been reported in chronic pain, anxiety, and depression, where excessive top-down control coexists with impaired sensory or emotional integration^19–21^.

The results highlight potential neural targets for intervention. Core nodes of the salience and interoceptive networks, such as the insula and ACC, may serve as biomarkers for symptom severity and treatment response. Interventions including non-invasive neuromodulation, mindfulness-based therapies, and interoceptive exposure interventions may help restore balance between cognitive control and bodily awareness^22,23^. Additionally, resting-state metrics such as ALFF and ReHo may serve as biomarkers to monitor treatment effects or identify individuals at risk for persistent somatic symptoms.

This study has several limitations that need to be considered. First, the cross-sectional design precludes causal inference; therefore, the directionality between altered brain activity and symptom severity cannot be determined. Second, rs-fMRI reflects hemodynamic signals rather than direct neural activity, and residual physiological influences (e.g., respiration and cardiac rhythm) cannot be fully excluded^24^, despite standard preprocessing and multiple-comparison correction. In addition, the moderate sample size may have limited sensitivity to detect subtle effects. ReHo, ALFF, and fALFF capture overlapping but non-identical aspects of spontaneous brain activity, and their interpretation should be considered within these methodological constraints. Although multiple rs-fMRI metrics were examined, each analysis applied strict permutation-based FWE correction. Nevertheless, the findings across metrics should be interpreted as complementary evidence, and future studies with larger samples may further validate the robustness of these results. It should also be noted that several significant clusters were relatively small in spatial extent. Third, ROI-based correlation analyses, using mean values from group-level clusters, may introduce selection bias. Therefore, we also performed voxel-wise regression analyses across the brain with clinical scores, providing an independent approach and convergent findings in the precuneus and insula. In addition, the sensitivity analyses showed that only the left inferior frontal gyrus and left precuneus remained significant, whereas the right hippocampus and left insula did not survive the most conservative threshold. Furthermore, this study focused on group-level analyses in a clinically homogeneous SSD cohort with predominant chest pain and did not include independent validation or comparison groups. As a result, the specificity and generalizability of the observed neural patterns cannot be fully established. It remains unclear whether these alterations reflect chest-pain-specific SSD features, broader SSD mechanisms, or transdiagnostic emotion-somatic processes. Future studies using larger, multi-site samples, independent validation cohorts, and appropriate clinical control groups (e.g., multi-symptom SSD, anxiety disorders, or cardiopulmonary symptom controls) are needed to assess reproducibility and diagnostic specificity. Longitudinal and multimodal designs may further clarify causal mechanisms and treatment-related neural changes.

In summary, SSD with predominant chest pain is associated with aberrant spontaneous activity across interoceptive, limbic, and cognitive control networks. Integrating multiple rs-fMRI metrics, these findings suggest a potential link between disrupted interoceptive and emotion-body integration and somatic symptom burden, offering a conceptual framework for developing mechanism-informed neuropsychiatric interventions.

## Electronic Supplementary Material

Below is the link to the electronic supplementary material.

Supplementary Material 1

Supplementary Material 2

Supplementary Material 3

Supplementary Material 4

## Data Availability

The raw data supporting the conclusions of this article will be made available by the authors (Jin-Wei Qiang, dr.jinweiqiang@163.com), without undue reservation.
