# Supplementary material for: Functional brain abnormalities in patients with somatic symptom disorder presenting with chest pain: a resting-state fMRI study
Source: Sci Rep. 2026 May 16;16:22292. doi: 10.1038/s41598-026-51822-2 (PMC13376194; doi:10.1038/s41598-026-51822-2)
Supplement: Supplementary file 1 — Supplementary Material 1 [file 41598_2026_51822_MOESM1_ESM.docx]

Supplementary Table1. Head motion parameters and group comparisons

| Parameter | SSD | HC | p-value |
| --- | --- | --- | --- |
| Mean FD (mm) | 0.082 ± 0.031 | 0.078 ± 0.029 | 0.46 |
| Max translation (mm) | 0.78 ± 0.42 | 0.73 ± 0.38 | 0.52 |
| Max rotation (°) | 0.73 ± 0.25 | 0.70 ± 0.23 | 0.48 |

FD, framewise displacement.

Supplementary Table2. Partial correlation analyses controlling for anxiety (GAD-7) and depression (HAMD) scores

| Brain Region | Metric | Clinical Scale | r (uncorrected) | p | r (adjusted) | p (adjusted) |
| --- | --- | --- | --- | --- | --- | --- |
| Right hippocampus | ReHo | SSS | -0.36 | 0.006 | -0.32 | 0.012 |
| Left precuneus | fALFF | PHQ-15 | 0.36 | 0.008 | 0.34 | 0.010 |
| Left precuneus | fALFF | SSS | 0.37 | 0.006 | 0.33 | 0.011 |
| Left middle cingulate gyrus | fALFF | GAD  -7 | -0.30 | 0.035 | -0.26 | 0.064 |

Supplementary Table 3. Sensitivity analyses for FD and cluster-forming thresholds

| Condition | Main significant regions |
| --- | --- |
| FD < 0.2 mm | Left IFG, right hippocampus, left insula, left precuneus |
| Voxel-wise p < 0.005 | Left IFG, right hippocampus, left precuneus |
| Voxel-wise p < 0.0005 | Left IFG, left precuneus |

IFG, inferior frontal gyrus


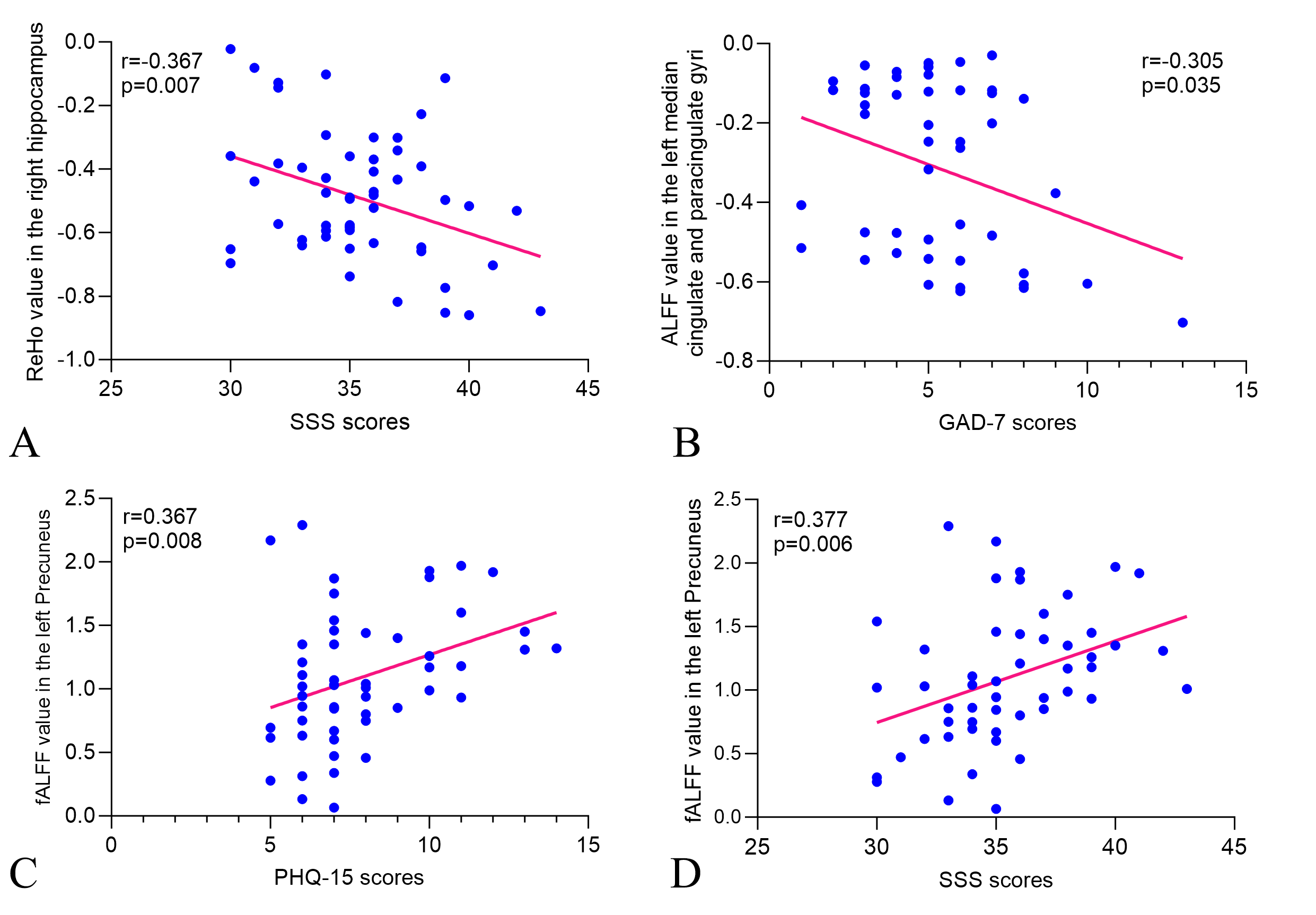


Supplementary Figure 1. Scatterplots showing significant correlations between resting-state fMRI metrics and clinical symptom scores in SSD patients.

(A) Negative correlation between ReHo in the right hippocampal and SSS scores; (B) Negative correlation between fALFF in the left middle cingulate gyrus , paracingulate gyrus and GAD-7 scores; (C, D) Positive correlations between fALFF in the left precuneus and PHQ-15/SSS scores, respectively. r = Pearson correlation coefficient; p = significance value; GAD-7, Generalized Anxiety Disorder-7; PHQ-15, Patient Health Questionnaire-15; SSS, Somatic Symptom Scale.
