# Supplementary figures and images for: Functional brain abnormalities in patients with somatic symptom disorder presenting with chest pain: a resting-state fMRI study
Source: Sci Rep. 2026 May 16;16:22292. doi: 10.1038/s41598-026-51822-2 (PMC13376194; doi:10.1038/s41598-026-51822-2)

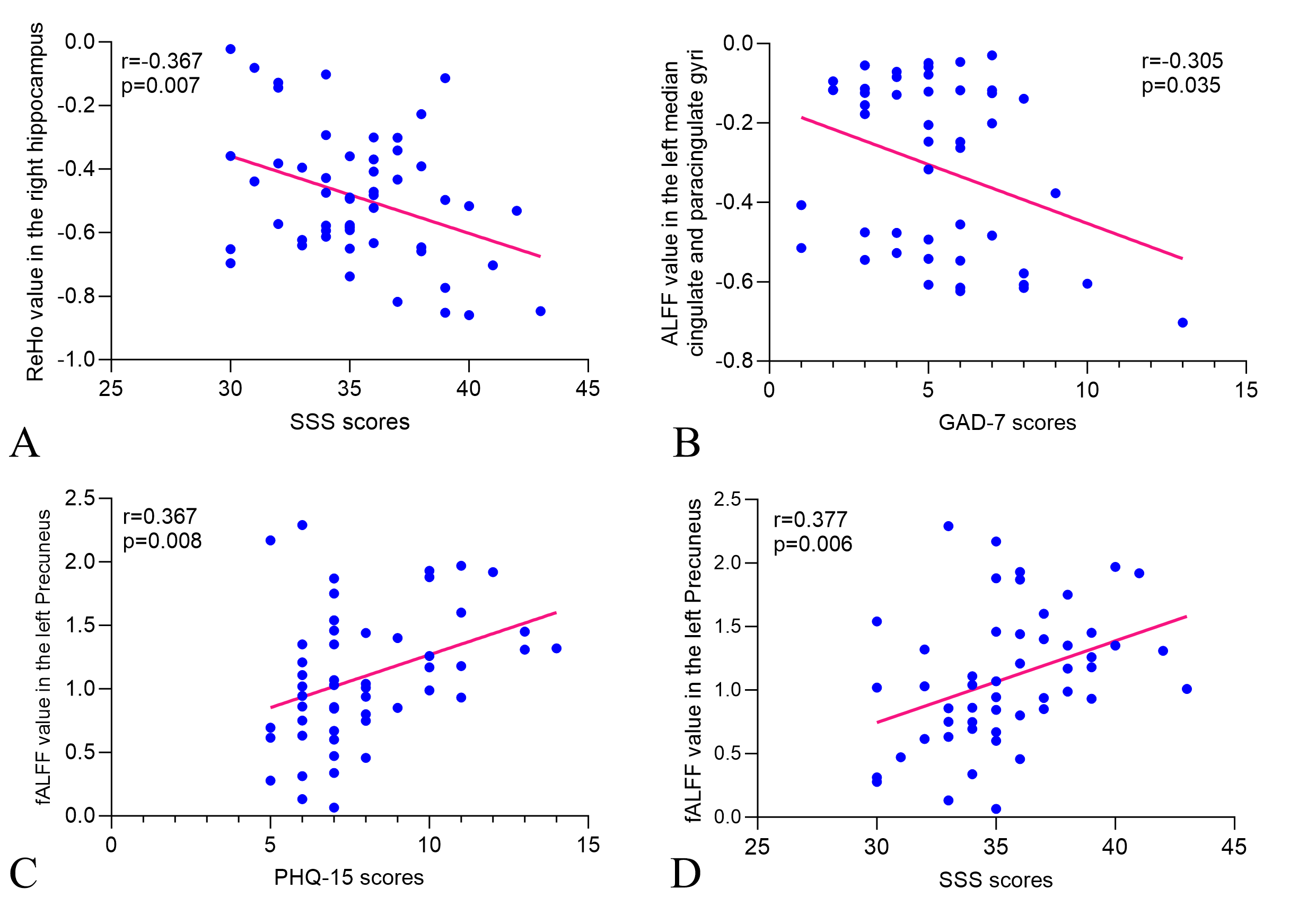

Supplement: Supplementary file 2 — Supplementary Material 2 [file 41598_2026_51822_MOESM2_ESM.tif]

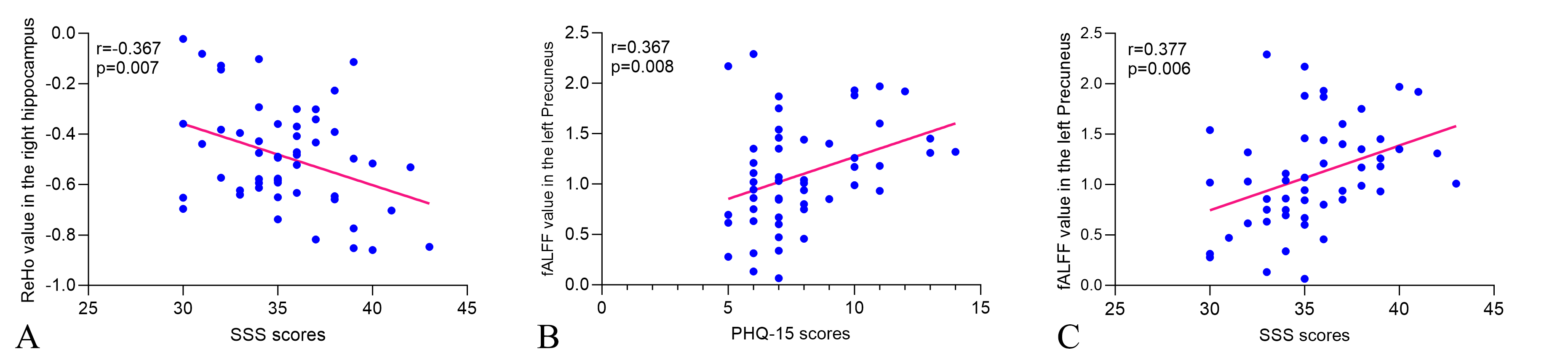

Supplement: Supplementary file 4 — Supplementary Material 4 [file 41598_2026_51822_MOESM4_ESM.tif]
